# An Immunological Perspective: What Happened to Pregnant Women After Recovering From COVID-19?

**DOI:** 10.3389/fimmu.2021.631044

**Published:** 2021-02-03

**Authors:** Sijia Zhao, Ting Xie, Li Shen, Hong Liu, Liling Wang, Xixiang Ma, Jianli Wu, Shuiqiao Yuan, Gil Mor, Aihua Liao

**Affiliations:** ^1^Institute of Reproductive Health, Center for Reproductive Medicine, Tongji Medical College, Huazhong University of Science and Technology, Wuhan, China; ^2^Department of Women’s Health Care, Maternal & Child Health Hospital of Hubei Province, Wuhan, China; ^3^Department of Obstetrics and Gynecology, Liyuan Hospital, Tongji Medical College, Huazhong University of Science and Technology, Wuhan, China; ^4^Department of Obstetrics and Gynecology, Tongji Hospital, Tongji Medical College, Huazhong University of Science and Technology, Wuhan, China; ^5^Department of Obstetrics and Gynecology, C.S. Mott Center for Human Growth and Development, Wayne State University, Detroit, MI, United States

**Keywords:** COVID-19, pregnancy, placenta, immune cells, recovery

## Abstract

The coronavirus disease 2019 (COVID-19) pandemic has been raging around the world since January 2020. Pregnancy places the women in a unique immune scenario which may allow severe COVID‐19 disease. In this regard, the potential unknown effects of severe acute respiratory syndrome coronavirus 2 (SARS-CoV-2) on mothers and fetuses have attracted considerable attention. There is no clear consistent evidence of the changes in the immune status of pregnant women after recovery from COVID-19. In this study, we use multiparameter flow cytometry and Luminex assay to determine the immune cell subsets and cytokines, respectively, in the peripheral blood and umbilical cord blood from pregnant women recovering from COVID-19 about 3 months (n=5). Our results showed decreased percentages of Tc2, Tfh17, memory B cells, virus-specific NK cells, and increased percentages of naive B cells in the peripheral blood. Serum levels of IL-1ra and MCP-1 showed a decreased tendency in late recovery stage (LRS) patients. Meanwhile, there was no significant difference in immune cell subsets in the umbilical cord blood. The placentas from LRS patients showed increased CD68^+^ macrophages infiltration and mild hypoxic features. The inflammatory damage of the placenta may be related to the antiviral response. Since the receptors, ACE2 and TMPRSS2, utilized by SARS-CoV-2 are not co-expressed in the placenta, so it is extremely rare for SARS-CoV-2 to cause infection through this route and the impact on the fetus is negligible.

## Introduction

The rapid international spread of coronavirus disease 2019 (COVID-19) has resulted in 54,558,120 confirmed cases and 1,320,148 deaths worldwide as of November 17, 2020 (1). COVID-19 is caused by severe acute respiratory syndrome coronavirus 2 (SARS-CoV-2), which is an enveloped, non-segmented, positive-sense RNA virus and belongs to the coronavirus family (2). The clinical manifestations may range from fever and cough to severe respiratory illness (3). In most cases, SARS-CoV-2 causes a mild or asymptomatic disease; however, in some cases, especially in elderly people, COVID-19 is life-threatening (4). One of the prominent features of severe SARS-CoV-2 infection is lymphopenia, which is accompanied by an uncontrolled release of proinflammatory cytokines called “cytokine storm”, leading to second ARDS attack and aggravating widespread tissue damage (5, 6). It is suggested that the susceptibility to COVID-19 is closely related to the individual’s immune status and immune dysfunction may play an important role in promoting the development of severe diseases.

Pregnancy is a unique immune state (7). On the one hand, mothers need to tolerate the fetus carrying paternal antigens rather than to reject them. On the other hand, it needs to maintain a certain anti-infection ability to prevent the invasion of pathogens. The immune system of pregnant women is a great challenge. The establishment and maintenance of pregnancy depend on the fine regulation of the maternal immune system. At different stages of pregnancy, the maternal immune state is not static but actively responds to the changes (7, 8). Whether pregnancy is an immunological advantage to control COVID-19 disease is still underdetermined. Moreover, the literature on this question is so far conflicting (9–11). Pregnant women represent a population at high risk of severe COVID-19 and are 1.5 times more likely to be admitted to an Intense Care Unit immediately after delivery than the nonpregnant peers (10). However, in a large, single-institution cohort study, 95% maternal infection at an initial presentation was asymptomatic or mild. SARS-CoV-2 infection during pregnancy was not associated with adverse pregnancy outcomes (11).

Another most concerning issue is whether vertical transmission of SARS-CoV-2 exists and whether it affects the fetus. Currently, evidence of vertical transmission of COVID-19 is extremely limited, and only a few studies have rigorously demonstrated its occurrence (12, 13). Several studies have demonstrated the presence of the virus in placental tissue (14–16). If the virus can reach the maternal-fetal interface, whether it can surpass the defenses of decidual immune cells and traverse the physical barrier formed by syncytiotrophoblasts to invade the fetus is a multifactorial event, which may be affected by viral load, coinfections, maternal immune response, and the presence of receptors that mediate the entry of SARS-CoV-2 (17, 18). Among these factors, the expression of receptors is the most fundamental premise. Although there is no conclusion on vertical transmission, infection during pregnancy may still have a certain impact on the structure and function development of the fetus and newborns due to the inflammatory response against the virus.

At present, few studies focus on the changes in the immune system after recovering from COVID-19, especially the lack of research on pregnant women after recovery. A previous study provided evidence of an inflammatory immune signature in the early recovery stage (ERS) of COVID-19, suggesting COVID-19 patients are still vulnerable after hospital discharge with both CD4^+^, CD8^+^ T cells, and naïve B cells decreased remarkably with highly expressed levels of inflammatory genes (19). Meanwhile, among the B cells, the plasma cells increased remarkably, whereas the naïve B cells decreased (19). A research team from the United Kingdom studied the memory T cells of 42 patients who recovered from COVID-19 and found that the breadth and intensity of T cell responses in severe patients were significantly higher than those in mild patients (20). Memory CD4^+^ T cells and CD8^+^ T cells were detected in 100% and 70% in patients who have recovered from COVID-19, respectively, with high titers of immunoglobulin G (IgG) antibodies which herald further protective immunity (21, 22).

We conducted this study to further clarify the immune status of pregnant women during the late recovery stage (LRS) of COVID-19. We analyzed the peripheral blood and umbilical cord blood from LRS pregnant women. Our results showed that the percentages of Tc2, Tfh17, memory B cells, virus-specific NK cells in the peripheral blood decreased, while the percentages of naive B cells increased. However, there is no significant difference in the immune cell subpopulations found in the cord blood. To a certain extent, the infection of pregnant women may not have a significant impact on the fetal immune system. The placenta of LRS women showed increased CD68^+^ macrophage infiltration and mild hypoxia. The inflammatory damage of the placenta may be related to the antiviral response. Since TMRSS2 is not expressed in the placenta of all stages of pregnancy, and also not co-expressed with ACE2, the probability of SARS-CoV-2 causing infection through the ACE2/TMRRSS2 route is extremely low.

## Methods

### Tissue Collection

All participants were recruited from the Department of Obstetrics and Gynecology at the Maternal & Child Health Hospital of Hubei Province, Wuhan, China. First-trimester placentas (n=10) (between 6 and 9 weeks of gestation) were obtained from the voluntary pregnancy termination (termination for nonmedical reasons). Second-trimester placentas (n=10) (between 15 and 24 weeks of gestation) were obtained from voluntary abortions after the fetus stopped developing. Placentas from both the NP group (normal term pregnancies) (n=8) and LRS group (n=5) were collected. We defined the LRS group as the date of nucleic acid turning negative to blood sampling is more than 14 days (19). All the enrolled patients suffered from mild to moderate disease. The criteria we used to categorize the severity of the disease based on the Guidelines for the Diagnosis and Treatment of COVID-19 Infection issued by the National Health Commission of the People’s Republic of China. The detailed information and basic characteristics of the subjects are summarized in **Table 1**. The study was approved and conducted according to requirements of the ethics committees at the Maternal & Child Health Hospital of Hubei Province, Tongji Medical College of Huazhong University of Science and Technology. Each participant signed a written informed consent form.

**Table 1:** Demographic details of women recovery from COVID-19.

	Patient 1	Patient 2	Patient 3	Patient 4	Patient 5
Age	30	30	28	43	35
G P	1 0	1 0	3 1	4 1	2 0
Gestation at admission	39w+1	36w+6	39w	31w+2	32w+5
Weight (g)	3800	2310, —	3490	1500	1850
Birth outcome	livebirth	livebirth*2	livebirth	livebirth	livebirth
Route of delivery	C-section	C-section	Vaginal delivery	C-section	C-section
Diagnosis date	Jan 26	Feb 6	Feb 13	Feb 25	March 2
Delivery date	Jan 27	Feb 8	June 12	June 9	July 6
Tissue collection date	PB	PB	UCB, placenta	PB, UCB, placenta	PB, UCB placenta
Blood collection date	June 3	June 3	June 12	June 9	July 6

PB, peripheral blood; UCB, umbilical cord blood.

### Multiparametric Flow Cytometric Analysis

Whole-blood samples were collected in heparin-treated tubes. Peripheral and umbilical blood monocyte cells were isolated by Ficoll-Hypaque density gradient centrifugation (Pharmacia). After washed twice by RPMI 1640 supplemented with 10% FCS (PAA laboratories), the cells were incubated with antibodies for 30 min at 4°C, then washed with staining buffer, and kept at 4°C until analysis. The experiments were performed according to the manufacturer’s instructions. Finally, total 75 immune cell subsets, including T cell subsets, NK cell subsets and γδT cell subsets, were determined by multiparametric FCM (**Figure S1**). The labeled cells were analyzed with BD FACSCantoTM flow cytometry (BD Biosciencces, San Jose, CA, USA). Data were analyzed in FlowJo version 10 software (Tree Star, Ashland, OR, USA).

### Cytokine Assay

Sera were separated by centrifugation and stored as aliquots at −80°C until analysis. Luminex liquid suspension chip detection was performed by Wayen Biotechnologies (Shanghai, China). The BioPlex protein array (Bio-Rad, Hercules, CA, USA) and Luminex 100 (Mirai Bio, Alameda, CA, USA) were used according to the manufacturers’ specifications. A cytokine panel (Bio-Plex Pro Human Cytokine Grp I Panel 27-plex, #M500KCAF0Y) was used to detect cytokines, including interleukin (IL)-1β, IL-1α, IL-2, IL-4, IL-5, IL-6, IL-7, IL-8, IL-9, IL-10, IL-12(p70), IL-13, IL-15, IL-17A, Eotaxin, IFN-γ, TNF-α, granulocyte–macrophage-colony stimulating factor (GM-CSF), Granulocyte colony-stimulating factor (G-CSF), monocyte chemotactic protein (MCP-1), macrophage inflammatory protein (MIP)-1β, MIP-1α, Vascular endothelial growth factor (VEGF), C-C Motif Ligand-5 (CCL5), interferon-inducible protein 10 (IP-10), Platelet-derived growth factor (PDGF-BB), basic fibroblast growth factor (Basic FGF). The lower limits of cytokine detection were different for each cytokine and experiment and usually ranged between 0.01 and 1.0 pg/ml.

### Histological Analysis

Placentas from both normal term pregnancies and women who recovered from COVID-19 (≥38 weeks of gestation) were collected and fixed in a 4% formaldehyde solution. Then, the samples were processed to paraffin blocks according to a standard procedure. Sections of 4 µm were cut and stained with H&E after being dewaxed and rehydrated.

### Immunohistochemistry and Immunofluorescence

Paraffin sections of these tissues with a thickness of 4 µm were dewaxed in xylene, rehydrated with graded ethanol, and then washed in PBS. Antigens were repaired by microwaving in 10 mmol/L citrate buffer at a pH of 6.0 (15 min). Endoperoxidase activity was blocked by 3% H2O2 in methanol, and nonspecific sites were blocked with 5% normal serum. The samples were then incubated with ACE2 (dilution 5 µg/ml; AF933; R&D Systems), TMPRSS2 (dilution 1:500; ab92323; Abcam), and CK7 (dilution 1:200;181598; Abcam), CD68 (dilution 1:1000; 28058-1-AP; ProteinTech), HLA-DR (dilution 1:300; 17221-1-AP; Proteintech) at 4°C overnight. After three washes with PBS, the sections were overlaid with the secondary antibody and developed in peroxidase substrate solution. The reaction was induced using 3,3-diaminobenzidine and counterstained with hematoxylin. Then, the sections were dehydrated through increasing concentrations of ethanol and xylene. Slides were created on 4 ∼ 6 random fields photographed at different magnifications to acquire images using an Olympus BX51 microscope and Olympus DP70 manager (Japan). The experiments were repeated 3 times.

### Quantitative Real-Time PCR

Total RNA was extracted with TRIzol reagent (Life Technologies, CA, USA) following the manufacturer’s instructions. An equal amount of total RNA (1 µg) was treated with gDNA Eraser reagent to eliminate potential genomic DNA and then used for cDNA synthesis (Takara Bio, Shiga, Japan). qRT-PCR amplification analysis was performed with 2 µl of cDNA using a Hieff qPCR SYBR Green Master Mix (11198ES03, Yeasen, China) on Quantagene q225 (Kubo Technology, China). Relative mRNA levels to calibrator were computed using the 2-CT method and β-actin was used for normalization. For PCR analysis, the following primers were used (5′-3′): β-actin forward, CTACCTCATGAAGATCCTCACCGA; β-actin reverse, TTCTCCTTAATGTCACGCACGATT; ACE2 forward, TTAATTTCTTTGTCACTGCACCTA; ACE2 reverse, CAGAAACTCTAGGCTGTTGTCA; TMPRSS2 forward, CCTGTGTGCCAAGACGACTG; TMPRSS2 reverse, TTATAGCCCATGTCCCTGCAG.

### Statistical Analysis

Statistical analyses were performed using the GraphPad Prism software, version 7. Data are presented as mean ± SEM. One-way ANOVA was used for multiple comparisons. Significance was defined as *P* < 0.05.

## Results

### Immune Profiles of NK and T, B Cell Subsets in the Peripheral Blood and the Umbilical Cord Blood of Pregnant Women After Recovery From COVID-19

**Figure 1:**
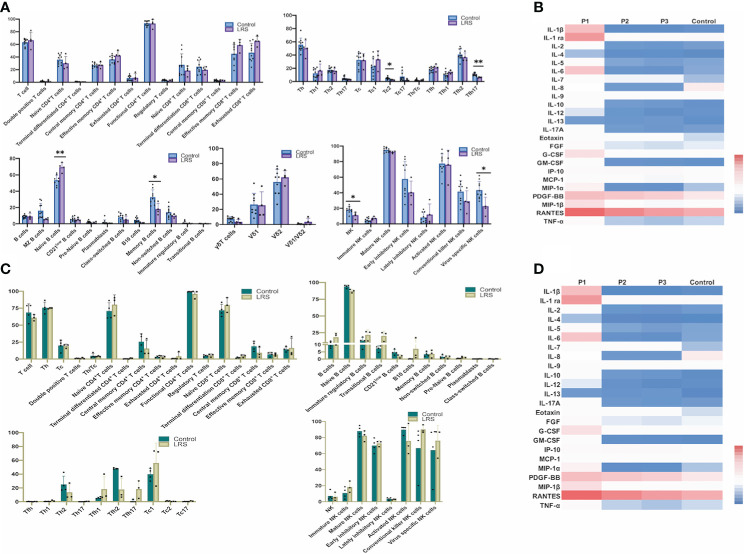
Immunological profiles of NK, T and B cell subsets and cytokine levels in the peripheral blood and the umbilical cord blood of pregnant women after recovery from COVID-19. **(A)** Multiparametric floow cytometry analysis of T-, NK-, and B-cell percentages of peripheral blood from pregnant women after recovery from COVID-19; **(B)** Luminex analysis of serum cytokine of peripheral blood of pregnant women after recovery from COVID-19. **(C)** Multiparametric flow cytometry analysis of umbilical cord blood; **(D)** Luminex analysis of serum cytokine of umbilical cord blood. Graphs were prepared using GraphPad Prism (Version 8.4.0).

Firstly, we analyzed the percentages of several lymphocyte subsets to get an overview of general distribution in the peripheral blood (obtained from four pregnant women recovered from COVID-19 and eight healthy pregnant women as the controls), mainly focusing on 75 immunological parameters including T cells, NK cells and γδT cells. The specific gating strategy of flow cytometry analysis is shown in **Supplementary Figure S1**. Our results showed decreased percentages of Tfh17 (CD3^+^CD4^+^CXCR5^+^CCR4^-^CXCR3^-^CCR6^+^) cells (*P* = 0.0045) in LRS women (**Figure 1A**). Tfh17 cells reside within the germinal centers (GCs) and play a major role in promoting B cell differentiation toward immunoglobulin-producing cells (23). CD8^+^ T cells can be classified into 2 major functionally different subsets (Tc1 and Tc2) based on the different cytokines they produce. Tc2 cells which exhibit reduced cytotoxic activity in comparison with Tc1 cells produce type 2 cytokine such as IL-4, IL-5, and IL-13, but not IFN-γ (24). Our results showed decreased percentages of type 2 (Tc2) cells (CD3^+^CD8^+^CXCR5^-^CXCR3^-^CCR4^+^) (*P* < 0.05) in LRS women (**Figure 1A**). The combination of CD19, CD21, CD27, CD38, IgM, and IgD was used to discriminate different B-cell subsets along their maturation pathway from precursors to effector cells (25) We observed increased percentages of naive B cells (CD45^+^CD19^+^CD27^-^IgD^+^) and decreased percentages of memory B cell subsets (CD19^+^CD27^+^CD38^-^) (**Figure 1A**). As for NK cell subsets, we observed decreased percentages of NK cells and decreased percentages of virus-specific NK cells (CD3^-^CD56^+^NKP46^+^) (**Figure 1A**). Heat map of cytokine levels from the whole blood also demonstrated an alteration in cytokines’ production (**Figure 1B**). Out of 27 cytokines and growth factors, the levels of IFN-γ and IL-15 were below the limit of detection and excluded from further analysis. Serum levels of IL-1ra and MCP-1 showed a decreasing tendency in the LRS group with statistically different from the controls (*P* < 0.05) (**Figure 1B**). MIP-1α tended to be lower in the LRS group than the controls but with no significant difference. Then we used multiparametric flow cytometry and Luminex assay to analyze the immune cell subpopulations and cytokines in the fetal cord blood and found no significant differences (**Figures 1C, D**).

### Immune Cell Infiltration and Histological Analyses of the Placenta After Recovery From COVID-19

**Figure 2:**
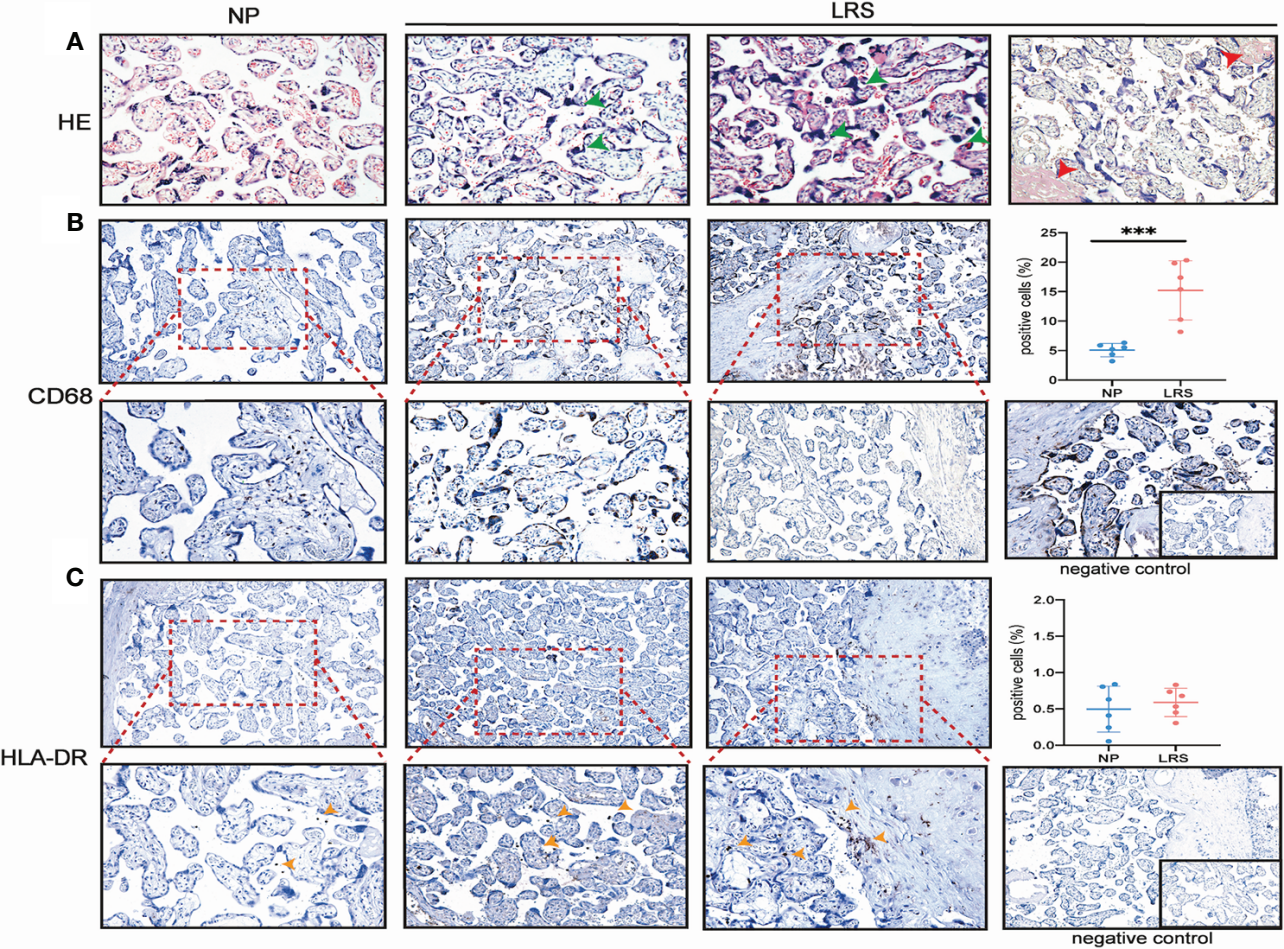
Histopathological analysis and immune cell infiltration in women recovery from COVID-19. **(A)** Morphology of placenta from the NP and LRS patients who were infected with SARS-CoV2 during the second trimester and gave birth after 3 months of complete recovery. The H&E staining showed mild hypoxic damages such as an increase in syncytial knots (green arrowhead) and maternal arteriole with atherosis and fibrinoid necrosis (red arrowhead) (H&E, 200×); **(B)** Increased CD68+ macrophages infiltration in LRS patients compared with controls (100× on the top; 200× on the bottom); **(C)** HLA-DR expression in the placenta of the LRS patients with no significant difference (100× on the top; 200× on the bottom). Graphs were prepared using GraphPad Prism (Version 8.4.0). NP, normal pregnancy; LRS, late recovery stage.

To compare whether there is a change in placental morphology and lymphocytes’ infiltration, we analyzed the placentas of pregnant women recovery from COVID-19 (**Figure 2**). The histological results showed that compared with the placentas of women who delivered normally, patients showed mild hypoxic damages such as an increase in syncytial knots (**Figure 2A**), which are thought to be developed in response to hypoxic damage or hypoxia-reperfusion injury (26). Since many factors can cause hypoxic damage to the placenta and the sample size was limited, it is uncertain that this change in the placenta was related to SARS-CoV-2 infection and requires further investigations. Immunohistochemical (IHC) staining for various leukocytes revealed an increased CD68^+^ macrophages infiltration (*P* = 0.0007), whereas the expression of HLA-DR, which was primarily regarded as a marker of activated T cells (27, 28), had no significant difference compared with the controls (**Figure 2B**).

### The Expression and Colocalization of TMPRSS2 and ACE2 at the Maternal-Fetal Interface

**Figure 3:**
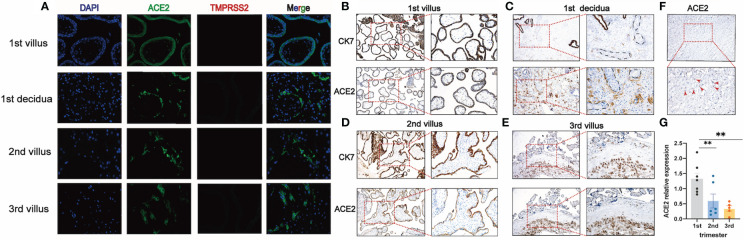
TMPRSS2 and ACE2 expression at different stages of pregnancy. **(A)** Immunofluorescence for DNA (DAPI, blue), ACE2 (green), and TMPRSS2 (red) in the placenta of first, second, third trimester (400×). No expression of TMPRSS2 was detected, so no co-expression of ACE2 and TMPRSS2 was observed; **(B, C)** Representative IHC images of serial sections of ACE2, CK7 expression in villi and decidua of 1^st^ trimester; **(D)** ACE2, CK7 expression in villi of 2^nd^ trimester; **(E)** ACE2, CK7 expression in villi of 3^rd^ trimester; **(F)** Expression of ACE2 in stromal chorionic cells in the chorionic plate (red arrowhead); **(G)** qRT-PCR analysis of the relative ACE2 mRNA levels in the first, second, third trimester; mean ± SEM, ** *P* < 0.01 by One-way ANOVA.

To clarify whether there exist vertical routes for SARS-CoV-2 entry, we used both the immunofluorescence and immunohistochemistry to detect the co-localization of ACE2 and TMPRSS2 in the placentas. Our results showed that no expression of TMPRSS2 was detected in early, middle, and late pregnancy by immunofluorescence (**Figure 3A**). To clarify the dynamic changes of ACE2 expression and localization in the first, second and third trimesters of pregnancy and which cell types express ACE2, we performed the co-staining of ACE2 and cytokeratin 7 (CK7) (a pan trophoblast marker) (29) in serial sections and chose the same field of view for observation (**Figures 3B–E**). In the first trimester, early chorionic villi had a complete outer layer of syncytiotrophoblasts and an inner cytotrophoblast layer. The blood vessels in the early placentas were not prominent. The expression of ACE2 was in the cytoplasm of both syncytiotrophoblasts and cytotrophoblasts and not in the anchoring villi column, which can differentiate into extravillous trophoblasts (EVTs) (**Figure 3B**). However, ACE2 was mainly expressed in the perivascular cells in the decidua, and little was expressed in the scattered EVTs, which can invade the interstitium (**Figure 3C**). In the second trimester, the terminal villi appeared with denser stroma surrounded primarily by syncytiotrophoblasts without a cytotrophoblast layer. ACE2 was expressed in some but not all the syncytiotrophoblasts (**Figure 3D**). In the third trimester, chorionic villi were highly vascularized to support the blood supply and nutrient exchange of the maternal-fetal circulation and only surrounded by a thin syncytiotrophoblast layer. ACE2 was abundantly expressed in EVTs of the decidua and almost not expressed in syncytiotrophoblast (SCTs) (**Figure 3E**). The stromal chorion cells in the chorionic plate, which form the fetal side of the placental disc, also expressed ACE2 (**Figure 3F**). Moreover, the expression level of ACE2 was highest in the first trimester, then gradually decreased, and was the lowest in the third trimester (**Figure 3G**).

## Discussion

We identified that most of the immune cell subsets in the peripheral blood of pregnant women recovering from COVID-19 returned to the normal level, with only a few subsets changed including the decreased percentages of Tc2, Tfh17, memory B cells, and virus-specific NK cells.

In the current study, the decreased percentages of total NK cells and virus-specific NK cells (CD3^-^CD56^+^NKP46^+^) were found. The molecule NKP46 is a major NK cell-activating receptor involving in the fight against bacterial, tumor, virus-infected cells, and mediating neutrophil apoptosis (30). A previous study reported a transient increase in the number of NKP46^+^ NK cells, followed by a continuous decrease in response to influenza (29).We found the number of pathologically activated NKP46^+^ NK cells decreased after recovery, which may indicate that NK cells are in the process of regaining homeostasis. Tfh17 (CD3^+^CD4^+^CXCR5^+^CCR4^-^CXCR3^-^CCR6^+^) belongs to circulating human memory Tfh cells (31). An elevated number of Tfh17 may play a role in maintaining humoral immune memory. Exhausted CD8^+^ T cells (CD3^+^CD8^+^PD-1^+^) showed an elevated tendency after recovery, which may be a hint left during infection that T-cell exhaustion curtailed T-cell responses leading to slow, subacute propagation of COVID-19 (9).

As previously reported, one of the prominent features of SARS-CoV-2 infection is lymphopenia which is associated with severe disease but is reversed when patients recovered (5). During normal pregnancy, the process of T and B lymphopoiesis will be moderately suppressed resulting in a reduction of lymphocytes (32, 33). However, in the current study, we did not find obvious evidence of lymphopenia compared with normal pregnant women. On the one hand, all mild to moderate cases were included, not severe cases. On the other hand, since we did not obtain the samples of these patients at the time of infection, it is difficult to evaluate the level of lymphopenia as the disease progresses. In contrast, no significant changes were found in the umbilical cord blood, indicating that maternal infection may have minimal impact on the fetal immune profiles. Although antibodies against SARS-CoV were tested positive from the maternal serum, breast milk, and umbilical cord blood, no viral RNA was present in the umbilical cord blood. Moreover, intravenous transplantation of hUC-MSC from umbilical cord blood has been confirmed by clinical trials to be a safe and effective method, which can be used as the priority for the rescue and treatment of severe COVID-19 (34). Although the immunological function of LRS women is still vulnerable, the immune cell subsets in the umbilical cord blood did not show abnormality which indicated that suffering COVID-19 in the middle or late trimester did not cause a significant impact on the fetal immune system.

A second key observation of our studies was the changes in the placentas of pregnant women recovering from COVID-19. We observed the increased CD68^+^ macrophages infiltration and mild hypoxic features (increased syncytial knots) in the placentas, although the patients had a normal delivery without other obstetric complications. A previous study focusing on the placentas from patients in the acute stage of COVID-19 had two common abnormalities: 1) vascular abnormalities leading to insufficient blood perfusion from mother to fetus, called maternal vascular hypoperfusion (MVM); 2) blood clots in the placenta are called interstitial thrombus and blood clots in the placenta called intervillous thrombi (35). However, the pathology of the placenta from patients who were infected in the first or second trimester and recovered from COVID-19 has not been addressed previously. Ng et al (36). demonstrated that the placentas from two women recovering from SARS in the first trimester were normal. Two women who had fully recovered from COVID-19 infection in the third trimester at the time of delivery, their placentas showed a larger area of avascular villi, with one of the two additionally demonstrating a large villous infarct (35). Currently, there is a lack of researches on the placenta after recovery from COVID-19, and accurate conclusions can only be drawn after expanding the sample size.

An additional major finding of the current study was the localization and expression profiles of ACE2 and TMPRSS2 in the placenta during pregnancy. Due to the above-mentioned changes in the placenta of convalescent women, the urgent question of whether SARS-CoV-2 can be vertically transmitted from a mother to the fetus and cause clinical infection of the fetus needs to be addressed. To enter the cell, SARS-CoV-2 uses its spike S protein to bind to the ACE2 receptors, which leads to the fusion with the cell membrane and endocytosis. Then, catalyzing the virus-cell membrane fusion is facilitated by proteolytic cleavage. TMPRSS2, a known human airway and alveolar protease, mediates the proteolysis of both protein S and ACE2, which can enhance the cell entry of both SARS-CoV and SARS-Cov-2 (37, 38). According to our results, TMPRSS2 was not expressed and not coexpressed with ACE2 in the placenta of all stages of pregnancy; thus, it is likely that SARS-CoV-2 cannot invade the fetus through this route. We also investigated the dynamic change of ACE2 expression in the placentas of normal pregnant women in the first, second, and third trimesters. Our results show that there is a relatively high expression of ACE2 at the human maternal-fetal interface of the first trimester, which is consistent with a previous study based on single-cell sequencing data available online (39). However, Pique-Regi et al (40). reported opposite results based on the same online dataset, ArrayExpress (E-MTAB-6701). This inconsistency may be due to the differences in the database construction and analysis methods. In the second trimester, the expression level of ACE2, which is expressed in some but not all syncytiotrophoblasts, decreased compared to that in the first trimester. Prior to our study, most studies only focused on the first and the third trimesters (39–43). There was a relative lack of data in the second trimester, and our research fills this gap. In the third trimester, ACE2 was abundantly expressed in EVTs in the decidua and almost not expressed in SCTs, but the overall expression level was lowest.

In summary, our data provide the immunological profiles of pregnant women who have recovered from COVID-19. We found the decreased percentages of Tc2, Tfh17, memory B cells, and virus-specific NK cells, and the increased percentages of naïve B cells existed in the peripheral blood of women recovery from COVID-19. The immunological function of LRS women is still vulnerable and it takes longer to return to normal. However, no significant difference in the immune cell subpopulations was found in the umbilical cord blood. The placentas from LRS women were infiltrated with the increased CD68^+^ macrophages and exhibited mild hypoxic features. The inflammatory damage of the placenta may be related to the anti-viral response. Since the receptors ACE2 and TMPRSS2, which are utilized by SARS-CoV-2, were not co-expressed in the placenta, we postulate that it is extremely rare for SARS-CoV-2 to cause infection through ACE2/TMPRSS2 route, thus its impact on the fetus is negligible. Nevertheless, further investigations are needed in a large sample size.

## Data Availability Statement

The original contributions presented in the study are included in the article/**Supplementary Material**. Further inquiries can be directed to the corresponding author.

## Ethics Statement

The studies involving human participants were reviewed and approved by Maternal & Child Health Hospital of Hubei Province, Tongji Medical College of Huazhong University of Science and Technology. The patients/participants provided their written informed consent to participate in this study.

## Author Contributions

SZ, TX, LS, and JW conducted sample collection. SZ performed the IHC experiments and RNA extraction. XM helped to optimize the IHC studies. SZ wrote the manuscript; HL, LW, SY, and GM participated in data interpretation and revision. AL contributed to the concept, design, text revision, and final approval. All authors contributed to the article and approved the submitted version.

## Funding

This work was supported by the Fundamental Research Funds for the Central Universities (2020kfyXGYJ116), and the Cultivation Plan of International Joint Research Platform of Huazhong University of Science and Technology (5001519009).

## Conflict of Interest

The authors declare that the research was conducted in the absence of any commercial or financial relationships that could be construed as a potential conflict of interest.

## References

[1] WHO Coronavirus Disease (COVID-19) Dashboard. Available at: https://covid19.who.int/ (Accessed November 17, 2020).

[2] Pal M Berhanu G Desalegn C Kandi V Severe Acute Respiratory Syndrome Coronavirus-2 (SARS-CoV-2): An Update. Cureus (2020) 12(3):e7426. 10.7759/cureus.7423 32337143 PMC7182166

[3] Guan W Ni Z Hu Y Liang W Ou C He J Clinical Characteristics of Coronavirus Disease 2019 in China. N Engl J Med (2020) 382:1708–20. 10.1056/nejmoa2002032 PMC7092819 32109013

[4] Hu B Guo H Zhou P Shi ZL Characteristics of SARS-CoV-2 and COVID-19. Nat Rev Microbiol (2020) 6:1–14. 10.1038/s41579-020-00459-7 PMC7537588 33024307

[5] Chen Z John Wherry E T cell responses in patients with COVID-19. Nat Rev Immunol (2020) 20:529–36. 10.1038/s41577-020-0402-6 PMC7389156 32728222

[6] Ragab D Salah Eldin H Taeimah M Khattab R Salem R The COVID-19 Cytokine Storm; What We Know So Far. Front Immunol (2020) 11:1446. 10.3389/fimmu.2020.01446 32612617 PMC7308649

[7] Aghaeepour N Ganio EA Mcilwain D Tsai AS Tingle M Van Gassen S An immune clock of human pregnancy. Sci Immunol (2017) 2:1–12. 10.1126/sciimmunol.aan2946 PMC5701281 28864494

[8] Mor G Aldo P Alvero AB The unique immunological and microbial aspects of pregnancy. Nat Rev Immunol (2017) 17:469–82. 10.1038/nri.2017.64 28627518

[9] Hanna N Hanna M Sharma S Is pregnancy an immunological contributor to severe or controlled COVID-19 disease? Am J Reprod Immunol (2020) 84:3–5. 10.1111/aji.13317 PMC7435498 32757366

[10] Ellington S Strid P Tong VT Woodworth K Galang RR Zambrano LD Characteristics of Women of Reproductive Age with Laboratory-Confirmed SARS-CoV-2 Infection by Pregnancy Status — United States, January 22–June 7, 2020. MMWR Morb Mortal Wkly Rep (2020) 69:769–75. 10.15585/mmwr.mm6925a1 PMC7316319 32584795

[11] Adhikari EH Moreno W Zofkie AC MacDonald L McIntire DD Collins RRJ Pregnancy Outcomes Among Women With and Without Severe Acute Respiratory Syndrome Coronavirus 2 Infection. JAMA Netw Open (2020) 3:e2029256. 10.1001/jamanetworkopen.2020.29256 33211113 PMC7677755

[12] Vivanti AJ Vauloup-Fellous C Prevot S Zupan V Suffee C Do Cao J Transplacental transmission of SARS-CoV-2 infection. Nat Commun (2020) 11:1–7. 10.1038/s41467-020-17436-6 32665677 PMC7360599

[13] Hosier H Farhadian SF Morotti RA Deshmukh U Lu-Culligan A Campbell KH SARS-CoV-2 infection of the placenta. J Clin Invest (2020) 130(9):4947–53. 10.1172/jci139569 PMC7456249 32573498

[14] Prochaska E Jang M Burd I COVID-19 in pregnancy: Placental and neonatal involvement. Am J Reprod Immunol (2020) 84:1–9. 10.1111/aji.13306 PMC7404599 32779810

[15] Algarroba GN Rekawek P Vahanian SA Khullar P Palaia T Peltier MR Visualization of severe acute respiratory syndrome coronavirus 2 invading the human placenta using electron microscopy. Am J Obstet Gynecol (2020) 223:275–8. 10.1016/j.ajog.2020.05.023 PMC7219376 32405074

[16] Patanè L Morotti D Giunta MR Sigismondi C Piccoli MG Frigerio L Vertical transmission of coronavirus disease 2019: severe acute respiratory syndrome coronavirus 2 RNA on the fetal side of the placenta in pregnancies with coronavirus disease 2019–positive mothers and neonates at birth. Am J Obstet Gynecol MFM (2020) 2(3):100145. 10.1016/j.ajogmf.2020.100145 32427221 PMC7233206

[17] DeRisi J Penland Lolita BPO Tyagi S Kramer FR Group NP DeRisi J Maternal HIV-1 viral load and vertical transmission of infection: the Ariel Project for the prevention of HIV transmission from mother to infant. Group (1996) 4:303–8. 10.1038/nm0798-822

[18] Gómez-Chávez F Cañedo-Solares I Ortiz-Alegría LB Flores-García Y Luna-Pastén H Figueroa-Damián R Maternal immune response during pregnancy and vertical transmission in human toxoplasmosis. Front Immunol (2019) 10:285. 10.3389/fimmu.2019.00285 30846989 PMC6393384

[19] Wen W Su W Tang H Le W Zhang X Zheng Y Immune cell profiling of COVID-19 patients in the recovery stage by single-cell sequencing. Cell Discov (2020) 6:31. 10.1038/s41421-020-0168-9 32377375 PMC7197635

[20] Peng Y Mentzer AJ Liu G Yao X Yin Z Dong D Broad and strong memory CD4+ and CD8+ T cells induced by SARS-CoV-2 in UK convalescent individuals following COVID-19. Nat Immunol (2020) 21:1336–45. 10.1038/s41590-020-0782-6 PMC7611020 32887977

[21] Ni L Ye F Cheng ML Feng Y Deng YQ Zhao H Detection of SARS-CoV-2-Specific Humoral and Cellular Immunity in COVID-19 Convalescent Individuals. Immunity (2020) 52:971–977.e3. 10.1016/j.immuni.2020.04.023 32413330 PMC7196424

[22] Grifoni A Weiskopf D Ramirez SI Mateus J Dan JM Moderbacher CR Targets of T Cell Responses to SARS-CoV-2 Coronavirus in Humans with COVID-19 Disease and Unexposed Individuals. Cell (2020) 181:1489–501.e15. 10.1016/j.cell.2020.05.015 32473127 PMC7237901

[23] Yan L de Leur K Hendriks RW van der Laan LJW Shi Y Wang L T follicular helper cells as a new target for immunosuppressive therapies. Front Immunol (2017) 8:1510. 10.3389/fimmu.2017.01510 29163552 PMC5681999

[24] Kemp RA Bäckström BT Ronchese F The phenotype of type 1 and type 2 CD8+ T cells activated in vitro is affected by culture conditions and correlates with effector activity. Immunology (2005) 115:315–24. 10.1111/j.1365-2567.2005.02168.x PMC1782161 15946249

[25] Clavarino G Delouche N Vettier C Laurin D Pernollet M Raskovalova T Novel strategy for phenotypic characterization of Human B lymphocytes from precursors to effector cells by flow cytometry. PLoS One (2016) 11:1–16. 10.1371/journal.pone.0162209 PMC5033467 27657694

[26] Stanek J Hypoxic patterns of placental injury: A review. Arch Pathol Lab Med (2013) 137:706–20. 10.5858/arpa.2011-0645-RA 23627456

[27] Saraiva DP Jacinto A Borralho P Braga S Cabral MG HLA-DR in cytotoxic T lymphocytes predicts breast cancer patients’ response to neoadjuvant chemotherapy. Front Immunol (2018) 9:2605. 10.3389/fimmu.2018.02605 30555458 PMC6282034

[28] Viallard JF Blanco P André M Etienne G Liferman F Neau D CD8+HLA-DR+ T lymphocytes are increased in common variable immunodeficiency patients with impaired memory B-cell differentiation. Clin Immunol (2006) 119:51–8. 10.1016/j.clim.2005.11.011 16413828

[29] Jost S Reardon J Peterson E Poole D Bosch R Alter G Expansion of 2B4+ natural killer (NK) cells and decrease in NKp46+ NK cells in response to influenza. Immunology (2011) 132:516–26. 10.1111/j.1365-2567.2010.03394.x PMC3075505 21214542

[30] Mandelboim O Lieberman N Lev M Paul L Arnon TI Bushkin Y Recognition of haemagglutinins on virus-infected cells by NKp46 activates lysis by human NK cells. Nature (2001) 409:1055–60. 10.1038/35059110 11234016

[31] Morita R Schmitt N Bentebibel SE Ranganathan R Bourdery L Zurawski G Human Blood CXCR5+CD4+ T Cells Are Counterparts of T Follicular Cells and Contain Specific Subsets that Differentially Support Antibody Secretion. Immunity (2011) 34:108–21. 10.1016/j.immuni.2010.12.012 PMC3046815 21215658

[32] Medina KL Smithson G Kincade PW Suppression of b lymphopoeisis during nomial pregnancy. J Exp Med (1993) 178:1507–15. 10.1084/jem.178.5.1507 PMC2191236 8228804

[33] Rijhsinghani AG Bhatia SK Tygrett LT Waldschmidt TJ Effect of pregnancy on thymic T cell development. Am J Reprod Immunol (1996) 35:523–8. 10.1111/j.1600-0897.1996.tb00052.x 8792935

[34] Shu L Niu C Li R Huang T Wang Y Huang M Treatment of severe COVID-19 with human umbilical cord mesenchymal stem cells. Stem Cell Res Ther (2020) 11:1–11. 10.1186/s13287-020-01875-5 32811531 PMC7432540

[35] Shanes ED Mithal LB Otero S Azad HA Miller ES Goldstein JA Placental Pathology in COVID-19. Am J Clin Pathol (2020) 154:23–32. 10.1093/ajcp/aqaa089 32441303 PMC7279066

[36] Ng WF Wong SF Lam A Mak YF Yao H Lee KC The placentas of patients with severe acute respiratory syndrome: A pathophysiological evaluation. Pathology (2006) 38:210–8. 10.1080/00313020600696280 PMC7131423 16753741

[37] Hoffmann M Kleine-Weber H Schroeder S Krüger N Herrler T Erichsen S SARS-CoV-2 Cell Entry Depends on ACE2 and TMPRSS2 and Is Blocked by a Clinically Proven Protease Inhibitor. Cell (2020) 181:271–80.e8. 10.1016/j.cell.2020.02.052 32142651 PMC7102627

[38] Heurich A Hofmann-Winkler H Gierer S Liepold T Jahn O Pohlmann S TMPRSS2 and ADAM17 Cleave ACE2 Differentially and Only Proteolysis by TMPRSS2 Augments Entry Driven by the Severe Acute Respiratory Syndrome Coronavirus Spike Protein. J Virol (2014) 88:1293–307. 10.1128/jvi.02202-13 PMC3911672 24227843

[39] Li M Chen L Zhang J Xiong C Li X The SARS-CoV-2 receptor ACE2 expression of maternal-fetal interface and fetal organs by single-cell transcriptome study. PLoS One (2020) 15:1–12. 10.1371/journal.pone.0230295 PMC7161957 32298273

[40] Pique-Regi R Romero R Tarca AL Luca F Xu Y Alazizi A Does the human placenta express the canonical cell entry mediators for SARS-CoV-2? Elife (2020) 9:3–7. 10.7554/eLife.58716 PMC7367681 32662421

[41] Valdés G Neves LAA Anton L Corthorn J Chacón C Germain AM Distribution of angiotensin-(1-7) and ACE2 in human placentas of normal and pathological pregnancies. Placenta (2006) 27:200–7. 10.1016/j.placenta.2005.02.015 16338465

[42] Neves LAA Stovall K Joyner JN Valdés G Gallagher PE Ferrario CM ACE2 and ANG-(1-7) in the rat uterus during early and late gestation. Am J Physiol - Regul Integr Comp Physiol (2008) 294:151–61. 10.1152/ajpregu.00514.2007 17977916

[43] Zheng QL Duan T Jin LP Single-cell RNA expression profiling of ACE2 and AXL in the human maternal-Fetal interface. Reprod Dev Med (2020) 4:7–10. 10.4103/2096-2924.278679

