# Supplementary material for: An Immunological Perspective: What Happened to Pregnant Women After Recovering From COVID-19?
Source: Front Immunol. 2021 Feb 3;12:631044. doi: 10.3389/fimmu.2021.631044 (PMC7886989; doi:10.3389/fimmu.2021.631044)

**Supplementary material**

**Fig.S1** Gating strategy for the detection of 63 immunological parameters by flow cytometric analysis.


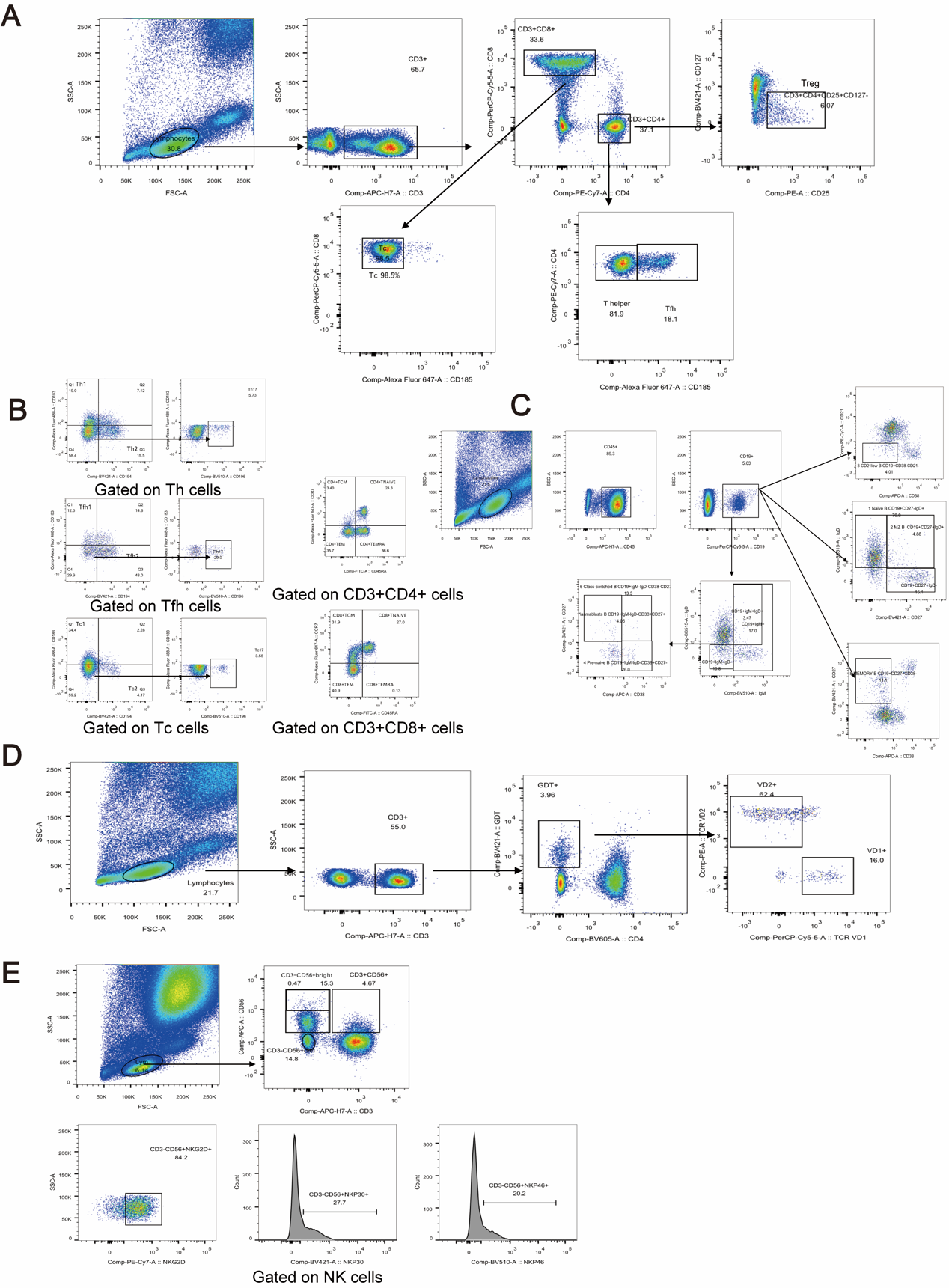

Supplement: Supplementary file 1 [file DataSheet_1.docx]
